# Estimating relative intensity using individualized accelerometer cutpoints: the importance of fitness level

**DOI:** 10.1186/1471-2288-13-53

**Published:** 2013-04-01

**Authors:** Cemal Ozemek, Heather L Cochran, Scott J Strath, Wonwoo Byun, Leonard A Kaminsky

**Affiliations:** 1 Clinical Exercise Physiology Program, Human Performance Laboratory, Ball State University, Muncie, IN, 47306, USA; 2 IU Health Ball Memorial Hospital, Cardiopulmonary Rehabilitation Program, 2401 University Ave, Muncie, IN, 47303, USA; 3 College of Health Sciences, University of Wisconsin-Milwaukee, PO Box 413, Milwaukee, WI, 53201-04133, USA

**Keywords:** Physical activity intensity, Physical activity assessment, Accelerometer

## Abstract

**Background:**

Accelerometer cutpoints based on absolute intensity may under or overestimate levels of physical activity due to the lack of consideration for an individual’s current fitness level. The purpose of this study was to illustrate the interindividual variability in accelerometer activity counts measured at relative intensities (40 and 60% heart rate reserve (HRR)) and demonstrate the differences between relative activity counts between low, moderate and high fitness groups.

**Methods:**

Seventy-three subjects (38 men, 35 women) with a wide range of cardiorespiratory fitness (maximal oxygen consumption (VO_2max_): 27.9 to 58.5 ml · kg^-1^ · min^-1^), performed a submaximal exercise test with measures of heart rate (HR) and accelerometer activity counts. Linear regression equations were developed for each subject to determine accelerometer activity counts for moderate and vigorous intensity physical activity corresponding to 40% and 60% of HRR. Interindividual variability of activity counts between subjects at both 40% and 60% of HRR was demonstrated by plotting values using a box and whisker plot. To examine the difference between absolute and relative activity cutpoints, subjects were categorized into 3 fitness groups based on metabolic equivalents (MET) (<10 MET, 10–13 MET, >13 MET).

**Results:**

At 40 and 60% of HRR, activity counts ranged from 1455–7520, and 3459–10066 counts · min^-1^, respectively. Activity counts at 40% HRR (3385 ± 850, 4048 ± 1090, and 5037 ± 1019 counts · min^-1^) and 60% HRR (5159 ± 765, 5995 ± 1131 and 7367 ± 1374 counts · min^-1^) significantly increased across fitness groups (<10 MET, 10–13 MET, and >13 MET, respectively).

**Conclusion:**

This study revealed interindividual variability in activity counts at relative moderate (40% HRR) and vigorous (60% HRR) intensities, while fitness level was shown to have a significant influence on relative activity counts measured at these intensities. Individualizing activity count cutpoints may be more representative of an individual’s PA level relative to their fitness capacity, compared to absolute activity count cutpoints.

## Background

Physical activity (PA) intensity levels can be defined by a value relative to one’s maximal aerobic capacity (i.e.,% VO_2_ reserve or % heart rate reserve [HRR]) or as an absolute value (i.e., metabolic equivalents [METs]). Moderate-intensity (MOD) using relative classifications is set at 40-60% of one’s HRR, whereas MOD intensity using absolute terms is set at 3–6 METs (1MET = 3.5 ml O_2_ · kg^-1^ · min^-1^) regardless of an individual’s aerobic capacity. Both definitions have been widely utilized to establish the health benefits of engaging in MOD to vigorous (VIG) -intensity PA across diverse populations [1-4].

In large scale epidemiologic studies, the absolute method of classifying PA has been used to establish associations between PA habits and various health outcomes, such as cardiovascular disease [5,6]. An issue with these absolute cutpoints is that they were originally developed assuming an average fitness level of 10 MET, which is not generalizable to all individuals [7]. On the other hand, early exercise training studies established relative thresholds of intensity that produced significant improvements in cardiorespiratory fitness and many health related measures [8-10]. Both have been used for a long period of time with absolute intensity used more so for large population analyses and relative intensity for exercise training studies.

The early identification of the benefits associated with high levels of PA was largely due to the use of self-report PA questionnaires. Many benefits and limitations exist with this method of PA monitoring. Most notably, these questionnaires can be administered in a cost and time efficient manner to a large number of people and many questionnaires have been demonstrated to be valid and reliable [11]. Limitations with self-report questionnaires stem from recall error that contributes to difficulties in quantifying frequency, duration and intensity [11,12]. However, reliable and accurate PA monitors appease limitations associated with subjective measures and have been incorporated in epidemiologic and clinical studies that provide insight into the health benefits associated with levels of PA [13-16]. This has enabled researchers to objectively classify PA intensity habits over a time period (i.e. 7–14 days). Additionally, the accelerometer’s increased reliability and user friendly interface have led to their widespread use in exercise based intervention research studies [17-22].

Accelerometers have the ability to differentiate varying levels of PA intensity through the measurement of accelerations that occur during ambulation which are quantified as activity counts [23]. Absolute activity count values previously developed by Freedson and colleagues [7] have been widely adopted as the standard cutpoints for classifying MOD and VIG intensities. These cutpoints were established by determining the association of accelerometer counts with absolute MOD and VIG intensity thresholds (i.e., 3–6 MET for MOD, >6 MET for VIG). Subsequently, in a similar fashion Miller et al. derived age specific cutpoints. Activity counts calculated by age specific (20–29, 40–49 and 60–69 years) equations were compared between age groups at MOD (40-59% VO_2max_) and VIG (>60% VO_2max_) intensities [24]. Their results indicated that at 40 and 60% of VO_2max_, younger individuals had higher relative activity counts when compared to older individuals. This suggests that using the standard 3–6 MET absolute intensity approach (1952–5724 counts · min^-1^) will underestimate PA intensity particularly for older (>60 yrs) age groups, while overestimating PA intensity in younger age groups.

Miller and colleagues provide valuable evidence demonstrating differences in activity count classifications when stratifying groups by age, but it is important to recognize that this difference may be due to age related declines in fitness (VO_2max_) [25-27]. The mean VO_2max_ of the 20–29 and the 60–69 year old groups in their study were 40.6 ± 6.5 ml · kg^-1^ · min^-1^ (~11.5 MET) and 30.1 ± 7.5 ml · kg^-1^ · min^-1^ (8.5 MET), respectively. In the current study we sought to extend Miller et al.’s method of creating absolute cutpoints relative to age by individualizing cutpoints relative to a subject’s fitness level. To date, no study has compared individually derived relative thresholds with the standard absolute thresholds. The purpose of this investigation therefore was twofold; (1) to demonstrate the interindividual variability of accelerometer activity counts measured at the 40 and 60% of HRR (MOD- and VIG-intensity, respectively), and (2) to compare individually derived activity counts between low, moderate and high fitness groups.

## Methods

### Subjects

Volunteers from Ball State University (BSU, n = 28) and the University of Wisconsin-Milwaukee (UWM, n = 45) read and signed an informed consent document, which was approved by the respective Institutional Review Boards prior to participating. To be included in this study, subjects had to be ≥18 years of age, free of any known cardiovascular disease and able to ambulate without any limitations. Exclusion criteria included individuals with any prior injuries or musculoskeletal conditions that limited their ability to perform the protocol, and those who were taking medications that may affect their heart rate (HR) response to exercise. The first visit was used to familiarize the subjects with the testing procedures. All subjects had their cardiorespiratory fitness measured during a maximal exercise test at a second visit. Then within 2 weeks, returned for a third visit to the laboratory to perform a standardized submaximal exercise test with simultaneous measurement of HR and accelerometry.

### Maximal exercise test

Prior to the second visit, subjects were given instructions to refrain from caffeine for ≥12 hours and strenuous activity for ≥24 hours before testing. Height was measured using a stadiometer and weight was measured using an electronic scale. Resting and exercise HR were measured using a Polar HR monitor (Polar Electro, Tampere, Finland). Oxygen consumption (VO_2_) was measured by a metabolic measurement system (True One 2400 Metabolic System, PARVO Medics, Sandy, UT), which was calibrated before each testing session. The BSU/Bruce Ramp protocol was used for the maximal exercise test at BSU, while the Bruce Protocol was performed by subjects at UWM [28,29]. It has been previously demonstrated that there is no significant difference in VO_2max_ measured between the two protocols [29]. The criterion for achieving VO_2max_ was a respiratory exchange ratio ≥ 1.1 and reaching volitional fatigue (rating of perceived exertion ≥ 18).

### Submaximal exercise test

Subjects were asked to abstain from caffeine/food consumption and exercise for at least 4 hours prior to the trial on the third visit. The exercise trial included 5-minute stages, beginning at 3.2 kilometers per hour (kph), and then increased to 4.8, 6.4, 8.0, and 9.6 kph with no resting periods between the stages. Submaximal testing was discontinued when the subject reached 85% of their maximal HR. The last 3 stages achieved prior to termination were selected for regression analysis [3.2, 4.8, 6.4 kph (n = 7); 4.8, 6.4, 8.0 kph (n = 8), and 4.8, 6.4, 9.6 kph (n = 58)]. During submaximal exercise, subjects’ HR were recorded during the last 10 seconds of every minute using a Polar HR monitor, in addition to recording accelerometer derived activity counts.

### Accelerometer

The ActiGraph GT1M (ActiGraph, Pensacola, FL) was used for the BSU subjects and the ActiGraph GT3X for subjects at UWM. The monitors were placed on the left hip at the waist midline with the knee. Previous research has shown no difference in activity counts between these two models [30,31]. The accelerometers were initialized with an epoch set to 60 seconds. In order for the accelerometers’ recorded activity counts to correspond with HR, accelerometer initialization time and submaximal exercise test clock time were synchronized.

### Data analysis

Data collected from the accelerometers were downloaded using ActiLife Software version 4.1.1 and exported into a Microsoft Excel file. The last 2 minutes of each submaximal stage were averaged to create a mean value to reflect activity counts for each stage interval. Heart rates during the last 2 minutes of each submaximal stage were averaged and used in calculating HRR% [(*HR*_*stage*_–*HR*_*rest*_)/(*HR*_*max*_–*HR*_*rest*_)100]. Relationships between HRR and accelerometer activity counts were examined using linear regression models. Intercepts and slopes were fitted for each individual including HRR% collected during submaximal exercise testing as the dependent variable and accelerometer activity counts during the same test as the independent variable.

All data are expressed as mean ± SD. Subjects were categorized into 3 fitness groups based on exercise capacity (<10 MET [n = 9], or 10–13 MET [n = 31], and >13 MET [n = 33]) in order to demonstrate the relationship between HRR% and accelerometer activity counts across different fitness levels. These categories represent very poor, poor to fair, and good and above fitness levels, respectively, as classified by data from the Cooper Institute [32] relative to the average age (26 years) of the subjects. The relationship between HRR% and activity accounts were plotted for all subjects and regression lines were generated for each respective fitness group. Furthermore, to compare the subjects’ relative activity count and Freedson’s cutpoints, differences between subjects’ relative (40 and 60% HRR) activity count and the respective absolute (1952 and 5725 counts · min^-1^) activity counts were determined. Subject characteristics and activity count between fitness groups were compared using an analysis of variance. Data analyses were performed using SPSS version 17 for Windows (SPSS Inc., Chicago, IL). For all statistical tests, a p < 0.05 was considered to be statistically significant.

## Results

Subject characteristics according to fitness grouping are presented in Table 1. Men’s VO_2max_ values ranged from 27.9 to 58.5 ml · kg^-1^ · min^-1^ and women’s ranged from 29.7 to 54.1 ml · kg^-1^ · min^-1^. VO_2max_ values differed significantly between each group with values of 31.6 ± 2.2, 40.1 ± 2.7, and 51.2 ± 3.7 ml · kg^-1^ · min^-1^ (low vs moderate, moderate vs high, low vs high fitness groups; all p < 0.001). Additionally, the moderate and high fit groupings differed significantly from the low fit group for weight and BMI. No significant differences were observed in mean resting HR or HR_max_ (p > 0.05) (Table 1).

**Table 1:** **Subject characteristics within groupings based off VO**_**2max**_**. Mean ± SD**

	**Low fit group**	**Moderate fit group**	**High fit group**
**n (#)**	9	31	33
**Male (#)**	4	11	23
**Age (y)**	28.8 ± 7.2	25.7 ± 5.7	25.8 ± 5.1
**Weight (kg)**	89.2 ± 22.6	73.3 ± 18.5*	72.0 ± 12.1*
**BMI (kg · m**^**-2**^**)**	30.2 ± 5.1	24.8 ± 4.6*	23.8 ± 3.0*
**VO**_**2max**_**(ml · kg**^**-1**^ **· min**^**-1**^**)**	31.6 ± 2.2	40.1 ± 2.7*	51.2 ± 3.7*^§^
**Resting HR (bpm)**	66 ± 7	62 ± 9	59 ± 8
**Max HR (bpm)**	189 ± 10	188 ± 8	188 ± 9

BMI = body mass index, VO_2max_ = maximal oxygen consumption, HR_max_ = maximal heart rate. Groups are separated by percentile rank of VO_2max_. Low fit group = <10 MET, Moderate fit group 10–13 MET, High fit group >13 MET. * Significant difference from low fit group, § significant difference from moderate fit group.

### Calculated activity counts at 40% and 60% HRR

**Figure 1:**
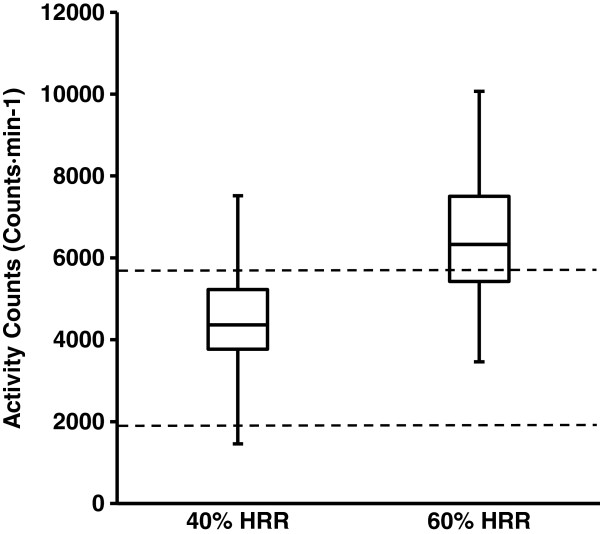
**Box plot of individual activity counts calculated at 40 and 60% HRR.** Dashed horizontal lines indicate absolute cutpoints for moderate (1927 counts · min^-1^) and vigorous (5725 counts · min^-1^) activity counts.

The inter-individual variability of accelerometer activity counts measured at the 40-60% of HRR is shown in Figure 1. The mean ± SD and range of activity counts of subjects at 40% HRR (MOD-intensity) were 4375 ± 1243 and 1455–7520 counts · min^-1^, respectively. For VIG-intensity (60% of HRR%) the mean ± SD activity counts 6451 ± 1592 counts · min^-1^ and ranged from 3459–10066 counts · min^-1^.

**Figure 2:**
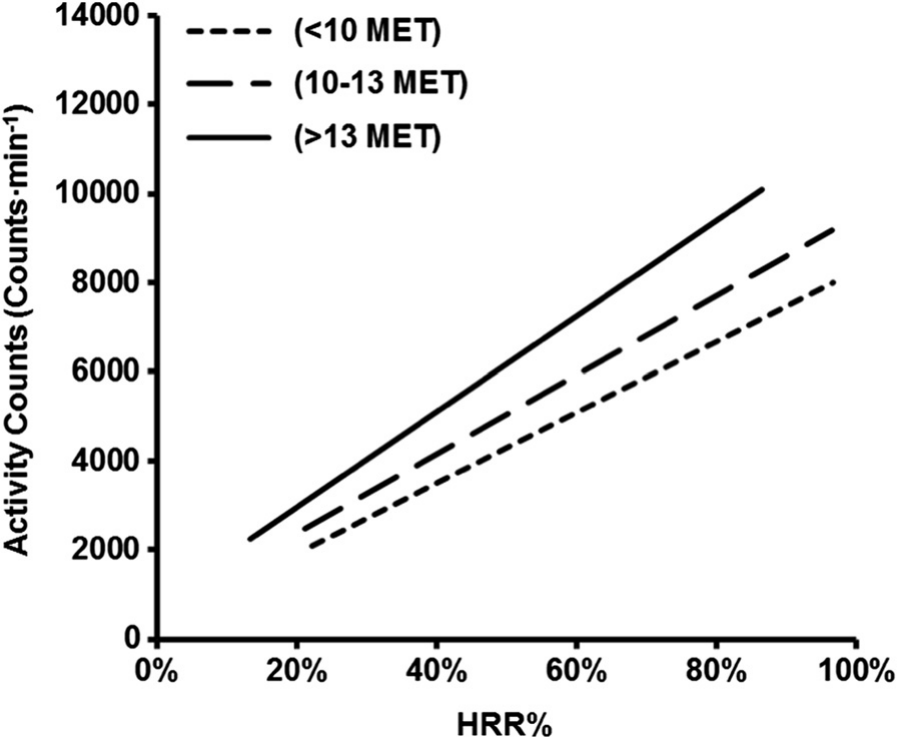
**Regression lines of activity counts at measured HRR% between fitness groupings.** Data collected during treadmill speeds of 3.2, 4.8, 6.4 kph, or 4.8, 6.4, 8.0 kph, or 4.8, 6.4, 9.6 kph. (<10 MET) low fit group, (10–13 MET) moderate fit group, and (>13 MET) high fit group.

Figure 2 presents differences in activity counts at varying intensity levels across fitness tertiles. Mean activity counts at 40% were significantly different between the low fit and high fit group (3385 ± 850 and 5037 ± 1019 counts · min^-1^, respectively; p < 0.001) and moderate fit (4048 ± 1090 counts · min^-1^) and high fit group (p = 0.001). Mean activity counts at 60% were significantly different between the low and high fit group (5159 ± 765 and 7367 ± 1374 counts · min^-1^, respectively; p < 0.001) and moderate fit (5995 ± 1131 counts · min^-1^) and high fit groups (p = 0.001).

Lastly, the difference between relative mean activity counts at 40% HRR and the absolute activity count cutpoint of 1952 counts · min^-1^ for MOD intensities across all fitness groups were; <10 MET: 1434 ± 850; 10–13 MET: 2097 ± 1090; and >13 MET: 3085 ± 1019 counts · min^-1^. Conversely, activity counts calculated at 60% of HRR for subjects in the low fit group (< 10MET) were on average lower by −545 ± 765 counts · min^-1^ compared to the absolute VIG cutpoint of 5725 counts · min^-1^. Activity counts calculated for subjects in the moderate and high fit groups were however greater than 5725 counts · min^-1^ by 270 ± 1131 and 1642 ± 1374 counts · min^-1^, respectively.

## Discussion

Our findings demonstrated that there was substantial interindividual variability in activity counts at both MOD- (~2000–7500 counts · min^-1^) and VIG- intensity (~3500–10000 counts · min^-1^) levels at 40 and 60% HRR (Figure 1). The data also revealed that fitness level influenced an individual’s activity count cutpoint at 40 and 60% HRR with both low and moderate fitness groups having significantly lower activity counts compared to the high fit group. These data verify that an individual’s fitness level is an important determinant of relative activity counts representative of MOD and VIG intensities.

Recent studies have explored if demographic characteristics such as age and body weight status may impact accelerometer derived activity count cutpoints of MOD and VIG intensities. Clear differences have been observed between the commonly used absolute cutpoints [7] and those derived specifically for different age groups [24] and for individuals with type 2 diabetes who are overweight and obese [33]). Certainly, applying absolute cutpoints specific to a population of interest may better represent relative PA levels compared to using generalized absolute cutpoints. However, due to potential variations in fitness level within these populations, even population specific absolute cutpoints may not represent the individual’s relative PA intensity levels.

The potential role of fitness can be considered in the recent study by Miller et al. which showed differences in activity counts at both MOD and VIG intensities between age groups. Their age group regression equations revealed lower activity counts for the older group compared to the younger group at MOD (45-59% VO_2max_: 2847–5376 counts · min^-1^ for 60–69 year olds and 4573–6786 counts · min^-1^ for 20–29 year olds) and VIG (≥60% VO_2max_: ≥5377 counts · min^-1^ for 60–69 year olds and ≥6787 counts · min^-1^ for 20–29 year olds) intensities [24]. These age based accelerometer cutpoints may provide a more accurate representation of one’s PA intensity compared to the current absolute cutpoints and are useful when retrospectively re-analyzing activity data originally classified using Freedson et al.’s absolute cutpoints. The association of decreases in activity counts with increases in age may have been due to age related changes in fitness capacity (VO_2max_), as suggested by Miller et al. Our findings support this concept as lower fit subjects had lower activity counts compared to higher fit subjects at a given relative intensity, regardless of age. Furthermore, age related fitness declines are evident in the cardiorespiratory fitness norms which show a decline in VO_2max_ with age (50th percentile for men of 44 ml · kg^-1^ · min^-1^ [12.5 MET] for 20—30 year olds compared to 29 ml · kg^-1^ · min^-1^ [8.3 MET] for 70–79 year olds) [32].

While VO_2max_ typically declines with age, there are interindividual cases that suggest otherwise and would therefore require individualized activity counts to better classify PA intensity as measured via accelerometry. One example from the current study would be to compare an older subject (38 years) with a VO_2max_ of 46.3 ml · kg^-1^ · min^-1^ (13.2 MET) to a younger subject (22 years) with a VO_2max_ of 31.5 ml · kg^-1^ · min^-1^ (9 MET). The MOD and VIG relative intensity activity count cutpoint was 500 and 1000 counts · min^-1^ greater for the older subject compared to the younger due to the older subject’s greater fitness level. Using Miller et al.’s age group equations would have effectively underestimated respective PA intensities for the younger subject while the older subject’s PA intensities would have been overestimated; however, individualized cutpoints would allow for a closer representation of PA intensity relative to fitness level.

This example brings to light the limitations of absolute intensity cutpoints when applied to a sample of subjects with ranging fitness levels from 27.0 to 58.5 ml · kg^-1^ · min^-1^. From this group of participants, 8 of the 9 low fit individuals’ (<10 MET capacity) relative activity counts (3833–5722 counts · min^-1^) calculated at 60% HRR did not achieve activity counts considered to be VIG by the absolute cutpoint 5725 counts · min^-1^, which would underestimate their time spent in VIG-intensity PA as defined by the absolute method. Similarly, underrepresentation of activity levels was demonstrated in a study by Stevenson et al. who reported that phase II cardiac rehabilitation patients did not spend any time in VIG PA when measured by traditional absolute cutpoints during a program session. However, during the same program sessions HR monitoring suggested patients were at relative VIG intensities (>60% HRR) [34]. Typical functional capacities expected of cardiac rehabilitation patients have been measured at 19.3 ± 6.1 (5.5 MET) in men, and 14.5 ± 3.9 ml · kg^-1^ · min^-1^ (4 MET) in women [35]. With such low aerobic capacities it would not be possible for patients (or any healthy older individual with a similar aerobic capacity) to reach absolute VIG-intensity accelerometer cutpoints even when at maximal efforts. Conversely, 29 of the 33 high fit individuals (>13 MET capacity) in our study achieved absolute activity counts of VIG-intensity, while most of these individuals met the absolute VIG criteria at only a relative intensity of 30-40% HRR. These individuals that reach an absolute level of VIG-intensity (>5725 counts · min^-1^) at lower relative intensities (<60% HRR) would have greater recorded time spent in VIG-intensity PA as defined by the absolute method.

In addition to creating absolute cutpoints relative to one’s age, Lopes and colleagues determined absolute cutpoints specifically for overweight and obese individuals with type 2 diabetes [33]. Similar to the current study, Lopes et al.’s subjects performed a treadmill calibration at 3 different speeds (2.5 km · hr^-1^, 5 km · hr^-1^, and 6 km · hr^-1^) while wearing an accelerometer. They showed the absolute activity count cutpoints for MOD- (3 MET) and VIG-intensity (6 MET) to be 1240 and 2400 counts · min^-1^ respectively; thresholds clearly lower than Freedson et al.’s proposed cutpoints for the respective intensities. Although they did not measure VO_2max_, Lopes et al. suggested that the lower cutpoints for MOD- and VIG-intensity were likely due to lower fitness levels commonly present in diabetic overweight and obese individuals. However, as with age, it is also known that fitness can vary within BMI ranges [36].

To demonstrate the importance of fitness related to developing cutpoints, we conducted ancillary correlations between activity counts at 40/60% of HRR with age, BMI, as well as VO_2max_. The correlation between fitness (VO_2max_) and activity counts were significant and explained 26% and 32% of the variability at 40 and 60% of HRR. However, less than 1% of variability (r^2^ = 0.0013 – 0.0034) in activity counts was explained by the individuals’ age and BMI. This suggests that previous absolute cutpoints based on age or BMI are less likely to account for differences in fitness level that may exist across age or varying body sizes. While, methods such as Miller et al.’s may be appropriate when used to describe a general population’s PA, they may misclassify an individual’s PA due to potential deviations in fitness (i.e. higher or lower) compared to an individual of similar age and gender. Our data best represents this concept as our cohort had a wide range of cardiorespiratory fitness (27.9 to 58.5 ml · kg^-1^ · min^-1^) over a small age range (18 to 39 yr). When the proposed individualized cutpoints relative to fitness were compared to cutpoints based on age using Miller et al.’s criteria, the absolute average differences were 961 counts · min^-1^ for the MOD-intensity cut point and 1308 counts · min^-1^ for the VIG cut point; clearly demonstrating the importance of creating individualized cutpoints based on fitness.

These observations emphasize the value of a method that would allow for the generation of individualized activity cutpoints appropriate for their fitness level. The relationship between HRR% and activity counts can be derived by having subjects perform a short (<10 minutes) treadmill protocol with 2–3 different submaximal speeds (3 minutes/stage to ensure physiologic steady state is reached) while wearing an accelerometer and a HR monitor. These data would then be presented in a scatter plot and using the regression line (y = mx + b; y = activity count, x = HRR%) to determine the activity count levels corresponding to relative MOD- (40% HRR) and VIG-intensities (60% HRR). Laboratories that would find relative intensity PA important and have access to a treadmill, heart rate monitor and an accelerometer may be able to employ this method.

However, it is important to acknowledge that performing such a protocol may not be appropriate for all settings; such as large surveillance studies that provide and collect PA monitors through the mail. Furthermore, studies that quantify PA as an additional descriptive measure and not a primary point of interest may choose to dedicate valuable subject time towards performing a battery of tests that help address the study’s primary purpose. Conversely, this method would be important for studies that place high value on quantifying MOD-VIG-intensity PA. Particularly those that seek to determine if differences in time spent in MOD-VIG-intensity PA, quantified by absolute and relative thresholds, are associated with different health related outcomes.

This study however is not without limitations. There were uneven subject distributions between the fitness tertiles, specifically in the lowest (n = 9). Additionally, future studies need to test the validity of the proposed method for individualizing cut points. It is recommended that future studies validate this method against the gold standard method of PA-intensity classification; VO_2_ response to exercise. This can be accomplished by recording PA via accelerometry while collecting expired gases during treadmill walking and/or running. PA intensity recorded by the accelerometer using individualized cut points relative to fitness (VO_2max_, as assessed through maximal exercise testing) can then be compared to relative PA intensity classified by VO_2_ collected during the same workloads. After establishing this methods validity, future studies should conduct a 7-day PA collection period to compare time spent in PA intensities between relative and absolute cutpoints in a heterogeneous population (large age and BMI range) to effectively demonstrate the variability that may exist in recorded time spent in MOD- and VIG-PA. Lastly, while data from two different sites and two different accelerometers were collected, protocols were strictly followed by highly trained technicians and previous findings have confirmed a high level of agreement between GT1M and GT3X accelerometer [30,31].

Individually derived, relative intensity cutpoints, would more accurately classify relative PA intensity during PA across all fitness levels. Additionally, applying individualized cutpoints for MOD and VIG activity, researchers will be able to extend the compelling associations between time spent in PA intensities and risk for various diseases. Foremost, this approach has not been applied in prospective studies where relative intensity is of importance, leaving great potential to examine relationships between relative cutpoints and outcomes of interest.

## Conclusion

In conclusion, this study demonstrated interindividual variability of accelerometer activity counts at relative levels of MOD- (40% HRR) and VIG-intensity (60% HRR) activity as assessed by accelerometry. This simple and time efficient approach used in this study for determining individual activity count cutpoints for MOD- and VIG-intensity has the potential to provide researchers a more representative PA profile of individual’s, than the widely used absolute intensity cutpoints.

## Abbreviations

HR: Heart rate; HRrest: Heart rate at rest; HRmax: Heart rate at max; HRstage: Heart rate at a given stage; HRR: Heart rate reserve; kph: Kilometers per hour; MET: Metabolic equivalent; MOD: Moderate; VIG: Vigorous; VO2: Oxygen Consumption; PA: Physical Activity

## Competing interest

The authors declare that they have no competing interests

## Authors’ contributions

CO completed the data analysis, results interpretation and manuscript preparation. HC performed data collection at Ball State University, while SS collected and provided data at the University of Wisconsin-Milwaukee. WB was involved in the manuscript preparation and LK completed the ethics application, design of this project and was involved in manuscript preparations. All authors read and approved the final manuscript.

## Pre-publication history

The pre-publication history for this paper can be accessed here:

http://www.biomedcentral.com/1471-2288/13/53/prepub
